# Investigating the Influence of Visual Function and Systemic Risk Factors on Falls and Injurious Falls in Glaucoma Using the Structural Equation Modeling

**DOI:** 10.1371/journal.pone.0129316

**Published:** 2015-06-08

**Authors:** Kenya Yuki, Ryo Asaoka, Kazuo Tsubota

**Affiliations:** 1 Department of Ophthalmology, Keio University School of Medicine, Shinanomachi 35, Shinjuku-ku, Tokyo, Japan; 2 Department of Ophthalmology, the University of Tokyo, Graduate School of Medicine, 7-3-1 Hongo, Bunkyo-ku, Tokyo, Japan; Harvard Medical School, UNITED STATES

## Abstract

**Purpose:**

To investigate the relationship between visual function and the risks of falling and injurious falls in subjects with primary open angle glaucoma (POAG)

**Methods:**

Questionnaires were conducted in 365 POAG patients to assess history of falls and falls with injury and general patient health. Structural equation modeling (SEM) was used to investigate the relationship between visual function, as measured by a patient’s binocular integrated visual field and visual acuity (VA), general health and the risks of falling and injurious falls.

**Results:**

Among the 365 subjects, 55 subjects experienced falls in the past year. A significant difference was observed in worse-eye VA between the faller and non-faller groups (p = 0.03). SEM of fallers obtained a Root Mean Square Error of Approximation (RMSEA) of 0.035 and a Comparative Fit Index (CFI) of 0.99. The 95% confidence intervals (CI) of regression coefficients from this model suggested better VA and worse VA were significant risk factors for falling. Among the 55 fallers, 22 subjects experienced an associated injury. There was a significant difference in gender between the non-injurious and injurious faller groups (p = 0.002). SEM of injurious fallers obtained a RMSEA of 0.074 and a CFI of 0.97. In this SEM model, the 95% CI of regression coefficients suggested gender and average total deviation values in the lower peripheral visual field were significant risk factors for an injurious fall.

**Conclusions:**

This study suggests that worse-eye and better-eye VAs are associated with falls. Furthermore, patients with inferior visual field loss and females were found to be at greater risk of injurious falls.

## Introduction

Falls, in particular falls associated with injuries, are one of the most serious public health concerns for the elderly in the world [1,2]. About 30% of individuals, over the age of 65 years, fall at least once in a year; 10%–20% of these falls lead to injury, and 5%–6% result in fractures [2]. In 2010 alone, there were 21,649 fall deaths among people aged 65 and older in the United States [3]. Falls are not only associated with physical consequences but also associated with reduced quality of life such as depression [4], fear of falling [5], restricted activity [6], hospitalization [7], and subsequent admission to a nursing home [1].

Glaucoma is a leading cause of blindness in the world [8] yet there is still controversy regarding the link between glaucoma (and consequential visual field defects) and the risk of falling [9–14]. In 2013, the number of people aged 40–80 years with glaucoma worldwide was estimated to be 64 million, and is expected to increase to 76 million by 2020 and 112 million by 2040 [15]. Glaucoma has widely been reported to be associated with reduced qualify of life and daily activities [16–18] but it is debatable whether glaucomatous visual field loss is associated with falls or not [9–14, 19–21]. One recent report suggested that the risk of falling is associated with damage in the inferior relatively peripheral VF [19]. Nonetheless, the risk of falling is influenced by many factors, such as body-mass index (BMI), general health status, gender and age [22,23]. In addition, the risk factors associated with falling compared with injurious falling may be different. The purpose of this study was to investigate the relationship between visual function and falls or injurious falls in subjects with primary open angle glaucoma (POAG) while considering patients’ general function.

The influence of visual function, namely visual acuity (VA) and VF status, on the risk of falling has usually been investigated independently, which is not ideal since the two measurements are significantly correlated [24–27]. Consequently, in the current study, structural equation modeling (SEM) was used to investigate the relationship between visual function and the risks of fall and injurious falls. SEM can model a set of relationships among a large number of variables simultaneously, in which continuous, discrete and factor variables are included [28]. In the current study, the main variables of interest were stage of glaucoma and risk of falling or injurious falling.

## Methods

The study was approved by the Research Ethics Committee of the Keio University School of Medicine. Written informed consent was obtained from all subjects after explanation of the nature and possible consequences of the study. The study was performed according to the tenets of the Declaration of Helsinki.

### Study Design and Subject Enrollment

A total of 601 patients who visited Keio University Hospital (Tokyo, Japan), Iidabashi Eye Clinic (Tokyo, Japan) or Tanabe Eye Clinic (Yamanashi, Japan) between the period of May 2011 and November 2011 were screened for eligibility by means of an ophthalmic examination that included slit-lamp biomicroscopy, funduscopy, gonioscopy, intraocular pressure measurements with Goldmann applanation tonometry and VF examination with the Humphrey Field Analyzer (HFA, Carl Zeiss Meditec, Dublin, CA), using the 24–2 Swedish Interactive Threshold Algorithm Standard Strategy. Patients with ophthalmological diseases that could compromise VA or cause VF loss, such as cataract (except for insignificant senile change) were excluded. Patients with angle closure glaucoma, secondary glaucoma, age-related macular degeneration, diabetic retinopathy, and any fundus disease apart from POAG were also excluded. Subjects who could not walk alone, or walk with a cane were also excluded. The eligible age was restricted to patients older than 40 years and less than 85 years. The purpose and methodology of the study were explained to every patient who met the inclusion criteria, and all patients agreed to participate.

### Diagnosis of POAG

POAG was diagnosed on the basis of the presence of the following three findings: (1) glaucomatous optic cupping represented by notch formation, generalized enlargement of cupping, senile sclerotic disc or myopic disc, or nerve fiber layer defects confirmed by glaucoma specialists (K.Y., and S.T.; see Acknowledgements) on fundus examination; (2) typical glaucomatous VF defects, such as Bjerrum scotoma, nasal step, or paracentral scotoma, compatible with optic disc appearance; and (3) an open, non-occludable angle observed on gonioscopy.

### Questionnaire Regarding History of falls

All participants answered a questionnaire including the following questions (translated from Japanese):

1. Can you walk without assistance? (Yes/No)
2. Do you use a cane or any kind of walking aid? (Yes/No)
3. How long do you spend walking on average per day? (The number of minutes was recorded.)
4. Are you afraid of falling? (Not at all; Not much; Afraid; Very afraid)
5. Have you had any falls in the last year? (Yes/No) *
6. Have you been injured by a fall in the last year? (Yes/No)

In addition, the following demographic information was recorded: age, sex, height, weight, alcohol intake, smoking history, current illnesses, and medical history (including medications taken orally).

### Integrated Visual Field

**Fig 1:**
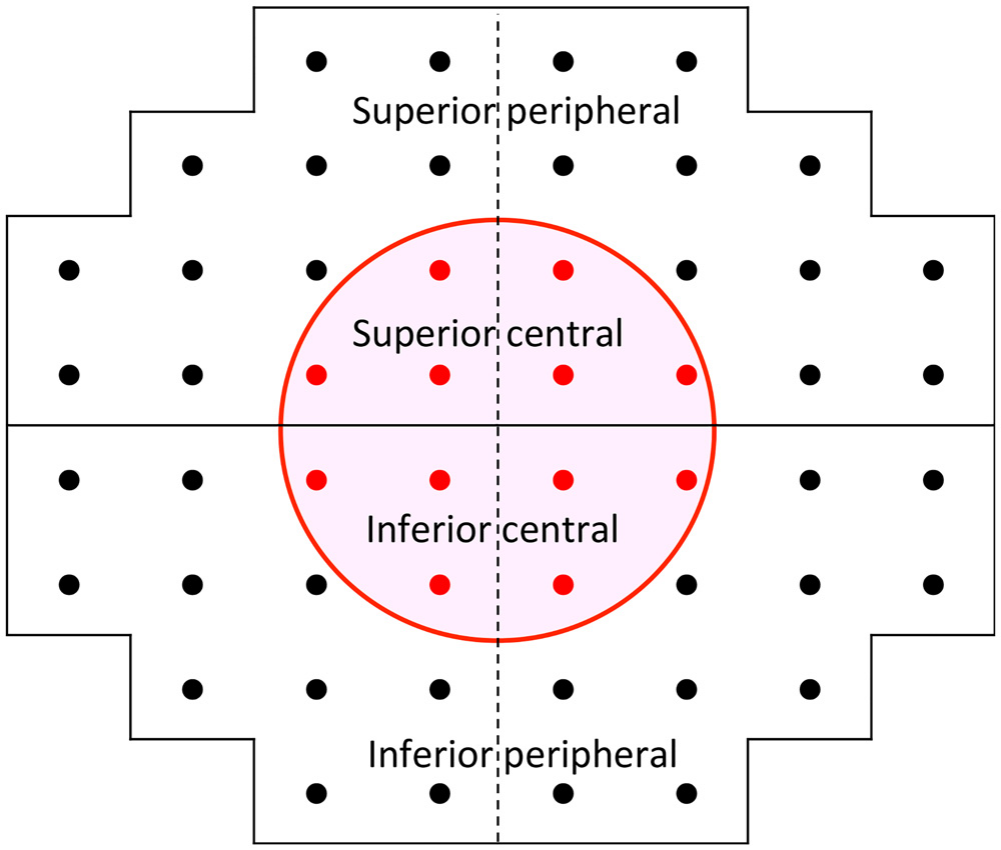
Mapping of the superior peripheral, superior central, inferior central and inferior peripheral areas. VF was divided outside and within central ten degrees. These areas also follow mapping in the 24–2 and 10–2 visual fields of the Humphrey Field Analyzer.

A binocular integrated VF (IVF) was calculated for each patient by merging a patient’s monocular HFA VFs using the ‘best sensitivity’ method [29–32]. In this method each total deviation (TD) value in the IVF is calculated by taking the maximum TD value from overlapping points in the monocular VFs, as if the subject was viewing binocularly. The IVF MD was then calculated as the mean of all 52 TD values across the IVF, while the means of TD values in the superior peripheral, superior central, inferior central and inferior peripheral areas were also calculated, following the areas indicated in Fig 1; thus, the VF was divided outside and within the central ten degrees (these areas follow the mappings in the 24–2 and 10–2 visual fields of the Humphrey Field Analyzer).

### Statistical Analysis

Structural equation modeling (SEM) was used to investigate the relationship between visual function, general function and the risks of falling and injurious falling. SEM is a multivariate technique well suited for testing the relationship between these variables and outcomes. It is one of the most frequently used statistical techniques in many social sciences, including psychological research, educational research, and market analysis. A considerable strength of SEM is that it can model ‘latent variables’, which are variables that cannot be measured directly, and are instead estimated in the model from several observed variables. In other words, observed variables are used as indicators of the latent variables. In SEM, statistical fit is often compared using the measures of Root Mean Square Error of Approximation (RMSEA) and Comparative Fit Index (CFI). An RMSEA in the range of 0.05 to 0.10 indicates a fair fit and values above 0.10 indicate a poor fit [33]. The CFI measurement is reported to be less affected by sample size than RMSEA [34] and a CFI value above 0.95 is indicative of a good fit [35].

In the SEM conducted here, the observed variables were: mean TD (mTD) in the superior peripheral VF, mTD in the superior central VF, mTD in the inferior central VF, mTD in the inferior peripheral VF, mTD in the whole field, better-eye VA, worse-eye VA, gender, age, BMI, complications of diabetes, complications of hypertension, usage of sleep aid, usage of sedatives and duration of walking in a day. The fitness of the SEM models was evaluated using the RMSEA and CFI.

All statistical analyses were carried out using the statistical programming language R (V.3.1.0, The R Foundation for Statistical Computing, Vienna, Austria). The R package ‘lavaan’ was used to carry out SEM. All of the variables were standardized to have a mean of 0 and a variance of 1, before analyses.

## Results

617 subjects were screened and 252 subjects were ineligible. The reasons for excluding subjects were as follows, where the numbers in parentheses indicate the number of subjects excluded: younger than 40 years old (28), older than 85 years old (25), refusal to participate (10), dementia (3), low VA (24), secondary glaucoma (62), glaucoma other than primary angle-closure glaucoma (16), history of retinal-detachment (21), diabetic retinopathy (36), bullous keratopathy (2), age-related macular degeneration (2), other ocular disease (1), subjects who could not walk unaided (0), and subjects who walk with a cane (16). A further six subjects were excluded because some of the answers the questionnaire were missing. As a result, 365 subjects were eligible and investigated in the current study.

Among the 365 subjects, 55 subjects experienced falls in the past year. The characteristics of non-fallers and fallers are shown in Table 1. There was a significant difference in the worse-eye VA of the faller and non-faller groups (p = 0.03, unpaired t-test).

**Table 1:** Comparison of variables between patients with and without a history of falls.

	fall+ (mean ± s.d.)	fall- (mean ± s.d.)	p value
Number	55	310	
Age	66.8 ± 11.5	64.0 ± 10.4	0.10
Better-eye VA (LogMar)	0.01 ± 0.03	0.00 ± 0.01	0.16
Worse-eye VA (LogMar)	0.03 ± 0.06	0.02 ± 0.04	0.03*
Gender (male:female)	29:26	181:129	0.46
BMI	22.4 ± 2.9	22.4 ± 3.0	0.99
Smoking status (Never/Previous/Current)	40/13/2	188/79/43	0.08
Alcohol intake (Never/Sometimes/Daily)	26/16/13	156/82/72	0.90
Prevalence of diabetes mellitus	12/55 = 21.8%	41/310 = 13.2%	0.10
Prevalence of hypertension	18/55 = 32.7%	93/310 = 30.0%	0.69
Sedative use	2/55 = 3.6%	8/310 = 2.6%	0.65
Sleeping aid use	1/55 = 1.8%	10/310 = 3.2%	0.99
Walking minutes per day	88.0 ± 104.4	84.2 ± 92.9	0.80
IVF MD (dB) [Range]	-2.0 ± 2.9 [-11.9–3.1]	-2.1 ± 4.0 [-18.5–5.3]	0.78
Better MD (dB) [Range]	-2.7 ± 3.4 [-14.5–2.2]	-3.0 ± 4.5 [-28.0–1.9]	0.53
Worse MD (dB) [Range]	-6.4 ± 5.8 [-24.5–0.8]	-7.6 ± 7.0 [-31.0–0.9]	0.19

Chi square test was used for the variable of gender, smoking status, alcohol intake, prevalence of diabetes mellitus, and prevalence of hypertension. Fisher's exact test was used for the variable of sedative use, and sleeping aid use. Unpaired t-test was used for other comparisons.

* represents p < 0.05.

**Fig 2:**
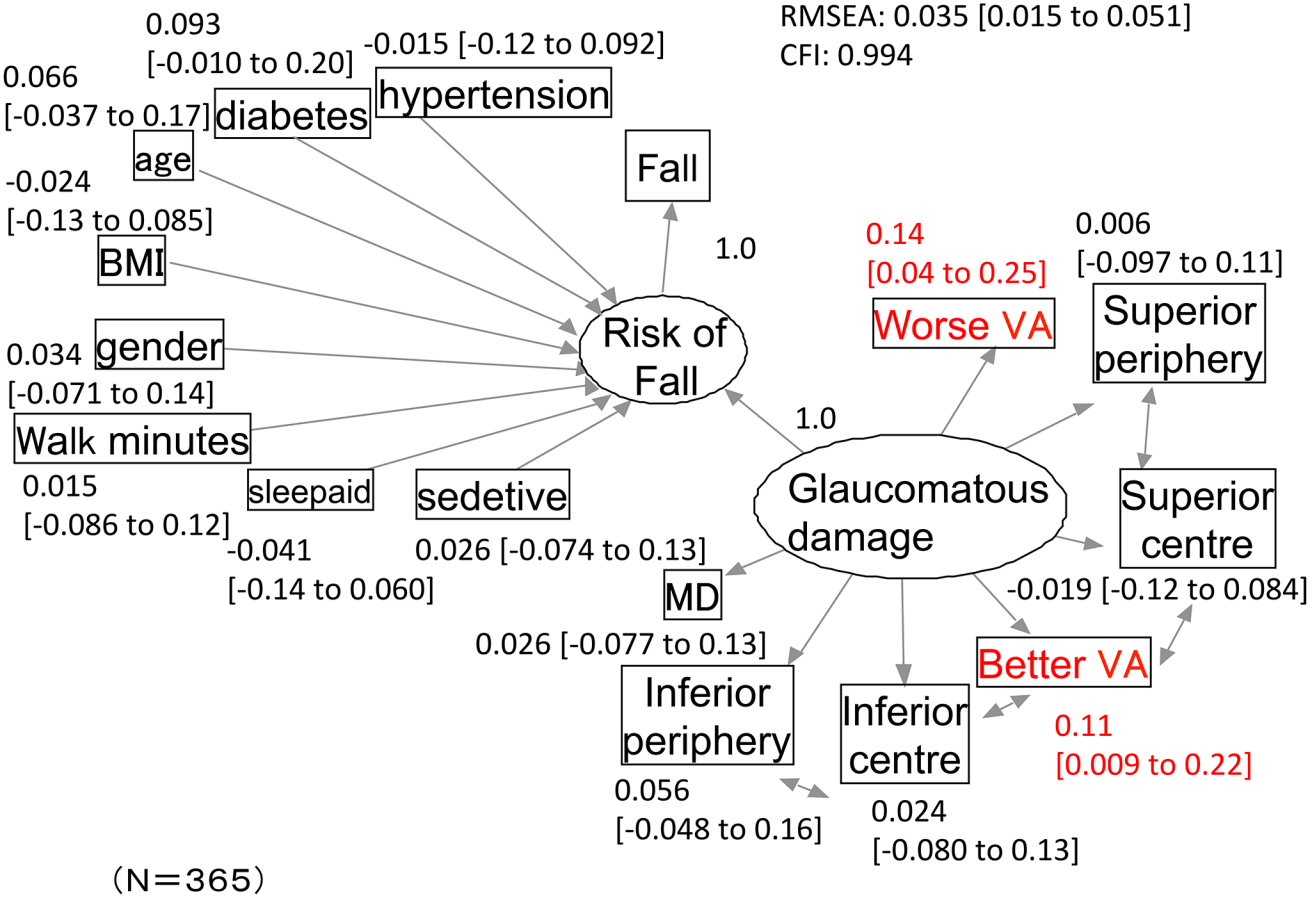
The structural equation modeling path diagram for the analysis of risk of falling. The model obtained an RMSEA of 0.035 and a CFI of 0.99. Among the 95% confidence intervals (CIs) of regression coefficients, only those from better-eye VA and worse-eye did not cross 0 (written in red). Variables in circles represent latent variables (‘Glaucomatous Damage’ and ‘Risk of falling’). Parentheses represent 95% CIs. BMI: body-mass index, gender: male: 0, female: 1, sleep aid: usage of sleep aid, sedative: usage of sedative. Superior peripheral, superior central, inferior central and inferior peripheral: means of the total deviation values in the superior peripheral, superior central, inferior central and inferior peripheral areas, respectively. MD: mean of the total deviation values in whole field. VA: visual acuity.

Abbreviations

s.d.: standard deviation, VA: visual acuity, LogMar: the logarithm of the minimum angle of resolution, BMI: body-mass index, MD: mean deviation, IVF: integrated visual field, TD: total deviation,

The path diagram describing the results of the risks of falling SEM analysis is shown in Fig 2. In the diagram, the circled variables represent the latent variables, ‘Risk of Falling’ and ‘Glaucomatous Damage’. The path diagram indicates that worse-eye VA, better-eye VA, mean TD values in the superior periphery, superior centre, inferior periphery and inferior centre regions, and mean TD value of whole field are determined by the level of glaucomatous damage. On the other hand, the risk of falling is determined by age, gender, BMI, usage of sleep aids and sedatives, minutes walked in a day, complications of diabetes, complications of hypertension, and the latent variable ‘Glaucomatous Damage’. This SEM model obtained an RMSEA of 0.035 and a CFI of 0.99. Only better-eye VA and worse-eye VA were statistically significant variables in the model; the mean and 95% confidence intervals (CI) of regression coefficients for these variables were 0.11 [CI: 0.009 to 0.22] and 0.14 [CI: 0.04 to 0.25], respectively.

Among the 55 fallers, 22 subjects experienced an associated injury. The characteristics of non-injurious fallers and injurious fallers are shown in Table 2. There was a significant difference in gender between the non-injurious and injurious faller groups (p = 0.002, chi square test).

**Table 2:** Comparison of variables between fallers with and without a history of injury.

	Fall with injury+ (mean ± s.d.)	Fall without injury- (mean ± s.d.)	p value
Number	22	33	
Age	68.0 ± 10.6	66.0 ± 12.2	0.54
Better-eye VA (LogMar)	0.01 ± 0.02	0.01 ± 0.04	0.52
Worse-eye VA (LogMar)	0.05 ± 0.07	0.02 ± 0.05	0.16
Gender (male:female)	6:16	23:10	0.002**
BMI	22.4 ± 2.9	22.4 ± 3.0	0.99
Smoking status (Never/Previous/Current)	19/3/0	21/10/2	0.17
Alcohol intake (Never/Sometimes/Daily)	13/4/5	13/12/8	0.27
Prevalence of diabetes mellitus	4/22 = 18.2%	8/33 = 24.2%	0.74
Prevalence of hypertension	8/22 = 36.4%	10/33 = 30.3%	0.64
Sedative use	1/22 = 4.5%	1/33 = 3.0%	0.99
Sleeping aid use	0/22 = 0.0%	1/33 = 3.0%	0.99
Walking minutes	76.6 ± 95.4	95.6 ± 110.7	0.50
IVF MD (dB) [Range]	-2.0 ± 2.9 [-11.9–3.1]	-1.6 ± 3.0 [-8.2–1.2]	0.28
Better MD (dB) [Range]	-2.9 ± 3.1 [-14.5–1.5]	-2.5 ± 3.6 [-8.0–2.2]	0.65
Worse MD (dB) [Range]	-7.4 ± 5.0 [-18.4–0.7]	-5.8 ± 6.3 [-24.5–0.8]	0.29

Chi square test was used for the variable of gender, alcohol intake, and prevalence of hypertension. Fisher's exact test was used for the variable of smoking status, prevalence of diabetes mellitus, sedative use, and sleeping aid use. Unpaired t-test was used for other comparisons.

** represents p < 0.01.

**Fig 3:**
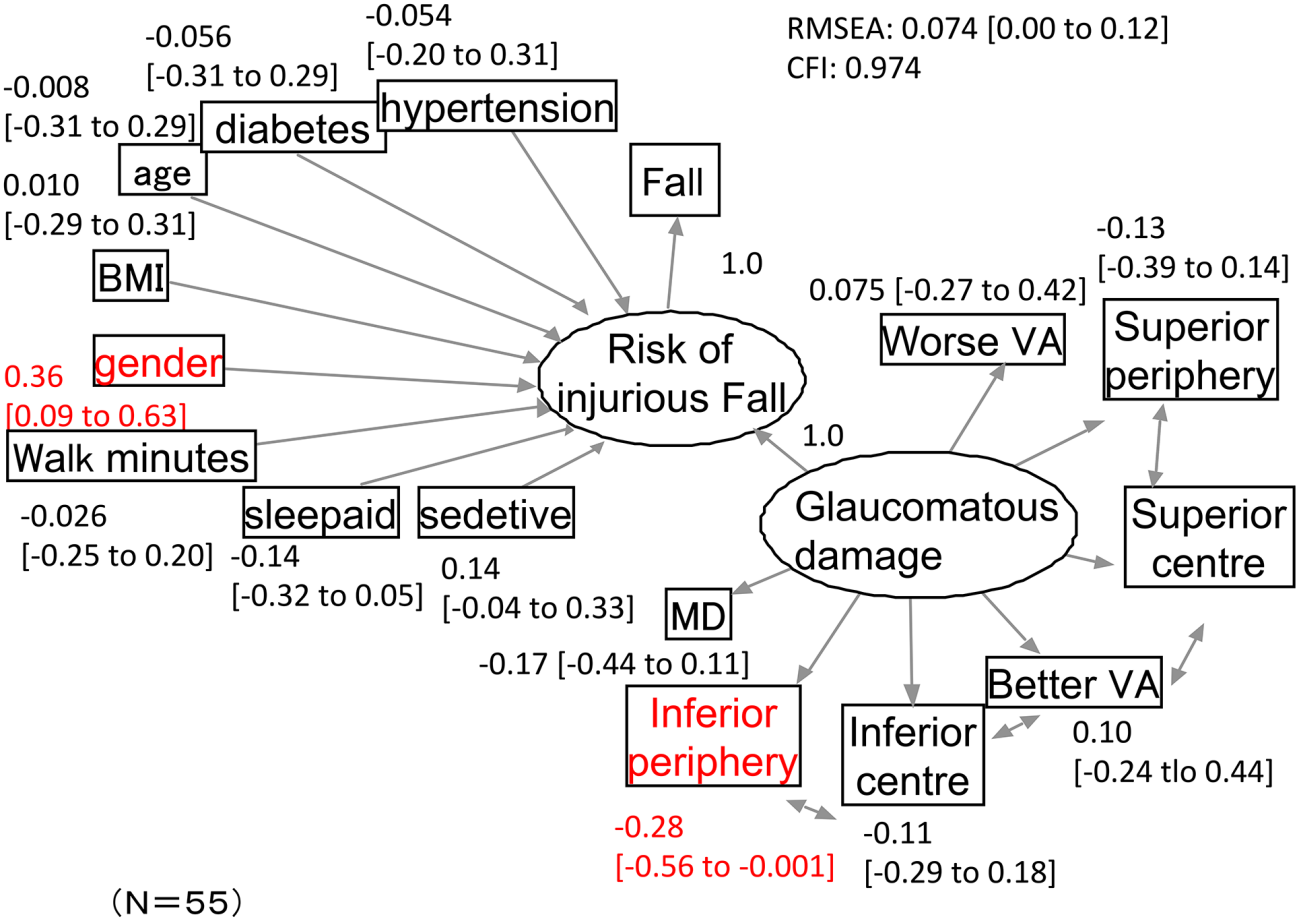
The structural equation modeling path diagram for the analysis of risk of injurious falling. The model obtained an RMSEA of 0.074 and a CFI of 0.97. Among the 95% confidence intervals (CIs) of regression coefficients, only those from gender and average TD values in the lower peripheral VF did not cross 0 (written in red). Variables in circles represent latent variables (‘Glaucomatous Damage’ and ‘Risk of injurious falling’). Parentheses represent 95% CIs. BMI: body-mass index, gender: male: 0, female: 1, sleep aid: usage of sleep aid, sedative: usage of sedative. Superior peripheral, superior central, inferior central and inferior peripheral: means of the total deviation values in the superior peripheral, superior central, inferior central and inferior peripheral areas, respectively. MD: mean of the total deviation values in whole field. VA: visual acuity.

Abbreviations s.d.: standard deviation, VA: visual acuity, LogMar: the logarithm of the minimum angle of resolution, BMI: body-mass index, MD: mean deviation, IVF: integrated visual field, TD: total deviation,

The SEM path diagram resulting from the analysis of injurious falls is shown in Fig 3. The SEM paths are identical to those shown in Fig 2, however, the magnitude of the coefficients are different. This model obtained an RMSEA of 0.074 and a CFI of 0.97. Only gender and the average TD value in the lower peripheral VF were significant variables in the model; the mean and 95% CI of regression coefficients for these variables were 0.36 [CI: 0.09 to 0.63] and -0.28 [CI: -0.56 to -0.001], respectively.

## Discussion

In this study, we have shown that best-corrected VAs in the better-eye and worse-eye of POAG patients are significantly associated with falls. A number of studies have reported that worse-eye VA is a risk factor for falls in the elderly [36,37]. In the EPIC-Norfork eye study, multivariable analysis examining the association between VA as a continuous variable and falls showed that a 1 unit increase in logMAR VA was associated with a 31% increase in the risk of a fall (OR = 1.3, 95% CI 1.0 to 1.7, p = 0.03) [38]. In the Singapore Malay eye study, unilateral severe VA impairment (log-MAR>1.0) in the worse-eye significantly increased the likelihood of a fall (multivariable adjusted OR = 1.6; 95% CI 1.1 to 2.3) [39]. In the Blue Mountains Eye Study, subjects with VA worse than 20/30 had 1.9 times higher risk of recurrent falls [40]. In a study of Osteoporotic fractures, women with declining VA had significantly greater odds of experiencing frequent falling during the subsequent year compared with women with stable or improved VA; odds ratios after adjustment for baseline VA and other confounders were 2.08 (95% CI 1.39–3.12) for loss of 1 to 5 letters, 1.85 (95% CI 1.16–2.95) for loss of 6 to 10 letters, 2.51 (95% CI 1.39–4.52) for loss of 11 to 15 letters, and 2.08 (95% CI 1.01–4.30) for loss of >15 letters [41]. All these results suggest that worse-eye VA is a prominent risk factor for falls in the elderly, supporting the results presented here. Pineless et al. also reported that subjects with disorders of binocular vision such as strabismus, amblyopia, and diplopia are 20% more likely to experience a fall [adjusted OR 1.20 (95%CI 1.18–1.21)] [42]. This result may also explain why visual acuity in the worse eye is associated with falls in the current study since it may deem a patient functionally monocular.

In subjects with glaucoma, however, no studies have shown that worse-eye VA is associated with falls. Haymes et al. examined the prevalence of falls in subjects with glaucoma and showed that glaucoma itself is associated with falls, but failed to show an association between worse-eye VA and falls [11]. Black et al. and Glyn et al. also showed no association between worse-eye VA and falls in glaucoma [9,19]. In our study, we have shown that VAs in the better eye and worse eye are associated with falls independent of VF severity. The reason for these inconsistencies is unclear, however one possible explanation is that previous studies evaluated the relationship between VA and VF independently, which is inappropriate because the measurements are inter-correlated.

In patients suffering injurious falls, we clearly showed that average TD values in the lower peripheral VF are associated with the risk of injurious falling. Previous reports have suggested an association between visual impairment and injurious falls [43,44], but there are a limited number of studies that investigated the association between injurious falls and VF loss. In the Los Angels Latino Eye Study, those with moderate to severe impairment in the peripheral VF were 1.40 (95% CI .94–2.05) times as likely to report falls with injury than individuals without any peripheral visual impairment; the trend was statistically significant (p = 0.04) [13]. Black et al. conducted a prospective study to evaluate the association between falls, injurious falls, and VF defects in subjects with glaucoma. They found that more extensive VF loss in the inferior region was associated with higher rate of falls with injury (RR, 1.80; 95% CI, 1.12 to 2.98) after adjustment for all other visual measures and potential confounding factors [19]. Our result are in agreement with these previous studies.

Vision is the only human sensory modality that provides us with information about distance, steps, ramps, and obstacles in our path. In one study, only fifty per cent of subjects with simulated severe VA loss (LogMar 1.65) could recognize a step down in their walking path compared with one hundred percent of subjects with simulated mild VA impairment (Logmar 0.88) [45]. Bochsler et al. evaluated the accuracy of target detection, such as a ramp up or down, while subjects were walking, and revealed that the accuracy of target recognition declines with lower VA [46]. Thus, it is possible that lower VA leads to a failure to detect obstacles, steps, and ramps, and a safe place to step, all of which could result in a fall.

It is also possible that subjects with inferior VF loss may fail to detect obstacles. While walking, fixation is gazed at the target where people are going [47]. People mainly use their peripheral VF to obtain information of obstacles while walking [48]. Marigold et al. evaluated head pitch angle and gait pattern changes with and without simulated inferior VF loss using glasses, while walking on a path with an irregular surface. As a result, it was suggested that downward head pitch angle increased and gait pattern altered when the lower VF was blocked [49]. These results suggest that information from the lower VF is important to guide lower limbs when walking in order to detect obstacles, and to find safe places to step. It is reasonable to hypothesize that eye-movement during walking may compensate VF loss in glaucoma; however, Vargas-Martin et al. evaluated the dispersion of eye movement in subjects with severe peripheral VF loss while walking, and found that eye-position dispersion becomes narrower, not wider, than in subjects with normal sight [50]. The authors speculated that the lack of compensation was due to the lack of stimuli in the area of VF loss. These results suggest that subjects with glaucoma with inferior VF loss may not be able to reduce the risk of falling using eye movements.

It is reported that VF loss is associated with reduced mobility and physical performance in elderly people [51] and in subjects with glaucoma [21,52]. Reduced mobility and physical performance is a significant risk factor for falling [53]. Black et al. examined the association between specific VF defects and functional status outcome measures in 74 community-dwelling older adults with open-angle glaucoma; outcome measures included physical performance tests (a 6-minute walking test, a timed up-and-go test and a lower limb strength test), a physical activity questionnaire (the ‘Physical Activity Scale for the Elderly’ questionnaire) and an overall functional status score. They found that inferior VF loss was associated with slower timed up-and-go scores, weaker lower limb strength and lower overall functional status scores. Superior VF loss was not associated with any of the functional status outcomes [54]. These results suggest that inferior VF loss make glaucoma subjects frailer than subjects with superior VF loss, or normal vision.

In this study, we also showed that female gender is significantly associated with injurious falls in subjects with glaucoma. Supporting this, female gender is reported to be a strong risk factor for falls in previous studies [44]. Black et al. reported that females are approximately three times more likely to have injurious falls than males after adjustment for age, visual factors, and possible confounders [19]. Patino et al. also reported that females are about 1.4 times more likely to have injurious falls than males [13].

The prevalence of falls in this study was 15.0% over the past one year. The prevalence is comparable with previous studies performed in Asian countries [55–57] and in Japan [58]. The prevalence rate of falls in our study and in Asian countries is lower than that of western countries where approximately 35–40% of generally healthy elderly people aged 65 or more experience a fall annually [59]. One possible explanation is a difference in living situations or marital status between Western countries and Asian countries. We intend to investigate this in future by asking patients about their living situation and marital status.

This study is subject to several limitations. First, it is a cross-sectional study, hence a future prospective study should be carried out in an attempt to obtain further evidence for the association between VA and falls / injurious falls. Second, the self-reported questionnaire “Have you experienced a fall in the past one year” may be a source of recall bias. However, self-reported falls have been used in a number of studies that evaluated the association between vision and falls [13,38,39]. Cummings et al. reported that it is most appropriate to ask elderly adults to recall falls over the preceding 12 months rather than a shorter period [60]. Another possible limitation is that we did not explore other systemic diseases, such as stroke, prior heart attack, angina, or use of anti-depressants as confounding factors in this study. Nor did we investigate whether a person with multiple falls is more likely to have a fall with injury. Furthermore, we did not investigate the nature of injuries resulting from a fall in the current study; however, in the Blue mountain eye study, Ivers et al. reported that subjects with glaucoma were 8 times more likely to have future hip fracture than subjects without glaucoma [61]. In addition, Coleman et al. reported that subjects with severe binocular visual field loss have a greater risk of hip fracture (HR = 1.66; 95% CI: 1.19–2.32) [62]. Loriaut et al reported that in a case-control study, 10 patients out of 96 subjects with hip fracture had glaucoma, but only 5 out of 103 patients without hip-fracture had glaucoma (OR = 10.65; 95% CI 2.21–51.3) [63]. White et al. also reported that glaucoma was a significant risk factor for future hip fracture in men and in women (HR = 1.9 and 1.3 respectively) in the leisure world cohort study [64]. These previous papers strongly suggest that glaucoma is a risk factor for hip-fracture but more research is needed to explore what type of visual field defect is a risk factor for hip-fracture. Finally, our study did not include a control group consisting of healthy subjects.

## Conclusion

In this study, we showed that VAs in the better-eye and worse-eye of subjects with POAG were associated with falls. We also showed that inferior VF loss and female gender are associated with injurious falls. These results will help clinicians to concentrate on these regions when managing patients, and offer appropriate advice if these areas are damaged.
